# Interprofessional Team-Based Learning: A Qualitative Study on the Experiences of Nursing and Physiotherapy Students

**DOI:** 10.3389/fpubh.2021.706346

**Published:** 2022-01-31

**Authors:** Jacqueline Mei-Chi Ho, Arnold Yu-Lok Wong, Veronika Schoeb, Alex Siu-Wing Chan, Patrick Ming-Kuen Tang, Frances Kam-Yuet Wong

**Affiliations:** ^1^School of Nursing, The Hong Kong Polytechnic University, Kowloon, Hong Kong SAR, China; ^2^Department of Rehabilitation Sciences, The Hong Kong Polytechnic University, Kowloon, Hong Kong SAR, China; ^3^School of Health Sciences (HESAV), HES—SO University of Applied Sciences and Arts Western Switzerland, Lausanne, Switzerland; ^4^Department of Applied Social Sciences, The Hong Kong Polytechnic University, Kowloon, Hong Kong SAR, China; ^5^Department of Anatomical and Cellular Pathology, State Key Laboratory of Translational Oncology, The Chinese University of Hong Kong, Shatin, Hong Kong SAR, China

**Keywords:** interprofessional education (IPE), interprofessional team-based learning, health care education and training, nursing education, physiotherapy education

## Abstract

Traditional discipline-specific training has limitations in facilitating inter-professional communication and collaboration. To address this issue, two local universities in Hong Kong launched an interprofessional team-based learning program to allow the undergraduate healthcare students to form teams and experience collaborative problem-solving. This study aimed to evaluate the experiences of nursing and physiotherapy undergraduates following interprofessional learning activities. Twenty-seven 3rd-year nursing and physiotherapy undergraduates were recruited through purposive sampling. Semi-structured interviews were conducted, and written feedback was solicited until data saturation was achieved. An inductive thematic analysis was used for the data, and each theme was mutually exclusive. The findings revealed the positive experiences of the students with this interprofessional learning activity. Three main themes emerged: (1) the process of interprofessional learning; (2) profession-related outcomes of interprofessional learning; and (3) patient-related outcomes of interprofessional learning. The study indicated that interprofessional team-based learning activities enhanced learning experiences of the students through interactive learning with other healthcare students. Experiences of relationships that are trustful and complementary allow students to develop confidence in knowledge transfer and in interprofessional collaboration, as well as in providing a holistic patient-centered care. These findings substantiate the importance and value of interprofessional learning in healthcare education.

## Introduction

Interprofessional education (IPE) has received an increasing attention and interest as a valuable learning experience for healthcare professionals (1). The IPE prepares healthcare professionals to share knowledge and experiences within a team and develop a better understanding of healthcare problems by integrating multiple perspectives. This teaching approach is crucial because future healthcare professionals will encounter increasingly complex health problems, and there is a growing number of vulnerable groups suffering from various diseases that require a team management (2) to bring about positive patient outcomes (3).

In the process of IPE, students can have positive learning experiences (Table 1), including effective communication and recognition of different professional values (10). Additionally, they can build trusting relationships among team members to develop insights into teamwork (11). Importantly, IPE can nurture leadership and negotiation strategies in handling conflicts, which has a sustainable effect on enabling students to maintain an effective team spirit after graduation (12). The ability of students to function as effective members of a team can facilitate interprofessional collaboration, which can ultimately optimize the quality of patient care (13).

**Table 1:** Description of studies on positive impact of interprofessional education in review.

**Ref no**	**References**	**Country**	**Methods**	**Participants/sample**	**Prevalence**	**Positive impact**
1	Black et al. (4)	US	Survey	*N* = 639 students (137 medicine, 127 pharmacy, 83 dental; 64 nursing; 60 physician assistant, 55 physiotherapy, 45 occupational therapy, 32 speech and hearing, 26 MPH, & 10 audiology)	Students' performance on Individual readiness assurance test (iRAT) increased over time [F_(_1, 9) = 4.35, *p* < 0.01]. A statistically significant association between reported team skills and team's average tRAT score (adjusted *R*^2^= 0.012; *p* = 0.04).	(1) Knowledge (2) Team work
2	Burgess et al. (5)	Australia	Survey	*N* = 311 students (222 medicine & 89 physiotherapy)	Eight-two to eighty-three percentage of the participants showed team tests assisted in their learning. Eighty-five percentage stated that this shared learning with other healthcare students can equip them to become a better team member. Eighty-six percentage showed satisfication to develop their clinical reasoning skills. Eighty-eight percentage respected different views from various professions.	(1) Equip for future work (2) Clinical reasoning skills (3) Learning attitude
3	Lochner et al. (6)	Italy	Mixed method	*N* = 33 students (occupational therapy & orthoptic)	Quantitative: Mean score of communication and team work skills increased (26.58–27.3); and interprofessional learning (34.24–34.27). Qualitative: Encourage communication and team-work skills	(1) Communication (2) Team-work skills
4	Pogge (7)	US	Survey	*N* = 30 students (14 osteopathic medicine & 16 pharmacy)	45.1–86.2% of the participants showed improved knowledge of the IPE content (*p* < 0.001). Eighty-eight percentage of them will recommended this course to other students. Eighty-eight to ninety-six percentage of the participants responded favorably on the enhancement of their learning.	(1) Knowledge (2) Learning attitude
5	Nakamura et al. (8)	Japan	Survey	*N* = 449 students (100 nursing, 95 medicine, 87 rehabilitation, 79 medical technology, 44 radiological technology, & 25 clincial engineering) Mean age: 22.2 ± 1.8	Four domains were significantly higher after the IPE (*p* < 0.05): understanding the role of each profession's specialization, regarding participation in group work; thought regarding the team in healthcare and welfare, feelings about cooperation among different professions.	(1) Roles (2) Team work (3) Team functioning in healthcare (4) collaboration
6	Wheeler et al. (9)	US	Survey	*N* = 169 students (104 medicine & 65 pharmacy) Male = 65 (38.5%) Nationality = 60.4% Caucasian, 24.9% Asian/Pacific, 4.7% Non-Hispanic black, 4.1% Hispanic, & 3.6% others)	Significant improvement in Interprofessional collaborative competencies attainment survey (*p* < 0.01) including communication, collaboration, roles and responsibilities, collaborative patient family centered appraoch; conflict management and resolution; and team functioning.	(1) Communication (2) Collaboration (3) Roles and responsibilities (4) Collaboration (5) Conflict management and resolution (6) Team functioning

Team-based learning can best facilitate an IPE, which is an evidence-based collaborative learning/teaching strategy involving multiple units of instructions, known as modules. Each module involved a three-step teaching cycle: (1) pre-class preparation, (2) in-class readiness assurance tests, and (3) an application-focused exercise. This approach can motivate students to adopt active learning through collaborative work with interprofessionals and enhance their ability in self-directed learning (14). The students can also achieve better communication through knowledge sharing, which enables them to gain new experiences. Nevertheless, healthcare professionals need to collaborate as teams to solve problems and to improve the patient outcomes. Team-based learning suits this expectation and enables students to apply their learning experiences to a real working environment (15).

In Hong Kong, most health professional curricula are based on traditional education, with a discipline-specific training model. Students have few learning opportunities to work with other healthcare disciplines, particularly across universities. To address this issue, two local universities, the Hong Kong Polytechnic University (HKPU) and the University of Hong Kong (HKU), piloted the Interprofessional Team-Based Learning for Health Professional.

Half-day onsite course of the students was priorly provided by reading materials that gives students from different disciplines the opportunity to collaboratively solve hypothetical clinical vignettes. The course was guided by a team-based learning model (16). This pilot course included undergraduate medicine, nursing, physiotherapy, and social work students. The details of this course have been reported elsewhere (2). The overall learning outcomes of this course were aimed at enhancing collaboration with students from other professions; comparing different roles, responsibilities, and professional limitations; making effective communication with respect to others; enhancing team relationships; developing collaborative work for the best interests of patients; recognizing stereotypical views; and recognizing different views from other professionals (17).

Nurses play a vital role in providing holistic care for patients, serve as communicators, and require coordination of patient care with different healthcare professions. Physiotherapists play a unique role in the care of patients in clinical settings. Their profession has increased awareness to be one of the integral members in the management of patients, which, in turn, needs to collaborate with other healthcare professionals. However, both disciplines still have traditional classroom training, which hinders their understanding of the importance of interprofessional collaboration. As there are limited studies exploring the learning experiences of nursing and physiotherapy undergraduate students regarding IPTBL, this study aimed to explore the learning experience and potential value following the IPTBL course.

## Methods

This study adopted a qualitative research approach using inductive thematic analysis to explore IPTBL experience among nursing and physiotherapy undergraduates. This approach is a useful and flexible tool that provides a detailed description of the data by identifying, analyzing, and reporting themes (18). The analysis framework in this study involved six steps: familiarization with the data, coding, searching for themes, reviewing themes, defining, and naming themes, and writing the story generated from the data (19). The data were reviewed from six focus groups with two to four students in each group, six personal interviews, three phone interviews, and two sets of written feedback. The participants could join either of these methods. This integration of multiple data collection methods led to an enhanced description of the IPTBL learning experience of the participants.

Two independent researchers read and re-read the data to familiarize themselves with it. Data were first coded according to the aim of this study, and then were further categorized and collated into theme clusters. The analysis was continued until data saturation was achieved, where no new concepts or themes has emerged. Repetitive checking of the data against transcripts was performed during the entire procedure to maintain the credibility of the study.

Briefly, all the participating students, irrespective of their discipline, were given an identical list of study materials 1 week in advance to prepare for the IPTBL course. When the students from the two universities attended the scheduled activities, they were assigned to teams of five to seven members, with each team comprising at least two disciplines. During the course, an in-class individual readiness assurance test (IRAT) and a team readiness assurance test (TRAT) were used to evaluate the readiness of the students, while a clinical scenario was presented as an application exercise for the students to tackle as individuals and as team members.

The inclusion criteria were nursing and physiotherapy undergraduates from HKPU who had participated in the IPTBL course (Table 2). A hundred and twenty participants were invited to voluntarily participate in the focus groups/interviews *via* email and follow-up phone calls. Seventeen nursing and ten physiotherapy undergraduate students in their 3rd year of study were interviewed between 2016 and 2017. All the participants had provided written informed consent before conducting interviews. The HKPU Research Ethical Committee approved this study.

**Table 2:** Design of the IPE course.

**Phrase**	**Session**
Preparation (1 week in advance)	Pre-class study materials from different disciplines delivered to students for preapration of the course
Implementation Face-to-face meeting	Session 1: Individual Readiness Assurance Test (iRAT) - Students worked individually on MCQs related to the reading materials
	Session 2: Team formulation & Ice-breaking Activity - Students formed into small teams with 5–7 members from at least two disciplines. - Students introduced each other for better understanding among themselves.
	Session 3: Team Readiness Assurance Test (tRAT) - Students worked as teams to discuss on the same set of questions. - Each team submitted one answer for each question.
	Session 4: Team Appeals - Students were allowed to appeal if they disagreed with the answers from the above session.
	Session 5 - Teachers as a role of facilitator provided constructive feedback to students.
	Session 6: Team Application Exercise - A clinical scenario designed to inspire team discussion. - Each team submitted one answer for each question.

A framework of open questions (Table 3) that explored the perceptions of the students of IPTBL guided the interviews. Research team members, who were not involved in the course teaching, recruited and interviewed the participants to allow them to freely express their learning experiences. Each interview lasted 30–60 min using a digital recorder. Data were first transcribed by research assistants and then verified by a researcher (JH). The transcripts were then independently read and analyzed by two researchers (JH and AW). Together with a senior researcher (FW), the team examined and identified themes. An in-depth discussion meeting was conducted to identify the initial inductive sub-themes that collapsed into higher order themes. Each theme was mutually exclusive. During the analysis, the coders discussed and reviewed the transcripts repeatedly to extract the key meanings. The results of the final analysis were obtained by discussion of the discrepant codes and when consensus was achieved among all team members.

**Table 3:** Interview question.

1. How would you describe your experience of participating in this course?
2. What is your experience of working in a team?
3. How do students from different professions work together to solve problems?
4. How could this learning experience help you in preparing for your future career?

## Results

Three main themes emerged: one involving the process and two related to the outcomes of the course. They are (1) the process of interprofessional learning, (2) the professional-related outcome of interprofessional learning, and (3) the patient-related outcome of interprofessional learning.

### The Process of Interprofessional Learning

Self-awareness of one's role in the related profession serves as an important element for engagement in interprofessional learning activities. The positive and active contributions of the students to the activity facilitated their peer learning and support. They also recognized their values in the team.

#### Participation and Involvement in the Discussion

The students recognized the differences in the background and professional knowledge of other team members. Their awareness of the importance of clinical partnership in their future work motivated them to acquire more specific knowledge of other disciplines that they had not yet encountered.

We went to a different school with different disciplines [for the IPTBL]. There were things that we did not know and had not encountered before. We may be in a working relationship when we meet again [in the future]. In view of this relationship, I understood that if I did not ask questions, I would not be told. I had to be more active. (361–366, N20)

The small group without a tutor facilitated a more vigorous discussion among us because the absolute answer was not given immediately, and this is what our future working environment will be: no teacher, no model answers, just wild guesses, and we will need to discuss and learn from each other. (5–7, S17)

#### Mutual Respect

The attitudes of the learners were positive during their interactions with the activity. The students understood that a harmonious learning environment could benefit from collaboration within the team. This kind of recognition creates a bonding relationship and encourages team members to reach consensus for conflict resolution.

We would discuss whether or not we agreed with each other. We also listened to each other's views. We might agree or disagree with each other, but the bottom line was that we accepted all views. I think we were all getting along and were very cooperative. (42–44, N15)

I think this experience is generated from your understanding of the other parties. Hence, one may learn to respect every profession and understand the nature of their work. This actually means that everyone has their own value in the team, and we cannot lose any of them. Again, everyone respects each other. I think I learned this. (163–167, S12)

Experience with this activity also helped the students learn how to use active listening and clinical reasoning skills when communicating respectfully with other team members.

I am better able to understand how they managed this case through observations. Furthermore, I have learned to respect their opinions. I am not saying that all the knowledge I have gained is correct, but I can listen more and develop my clinical reasoning. Then, I evaluate what is appropriate to say. Although doctors have a higher professional image, I do not think they always say the right thing. I do not think that is true based on my conversations with them. (69–73, S15)

#### Peer Learning and Support

The course design required the students to read the pre-class materials before the activity. During their discussion, this learning material served as a useful tool, stimulating the students to support one another for peer learning.

Some of the nursing students studied [a pre-class video], which gave them a better understanding of physiotherapy. Then, they asked me about the content of that video to clarify some concepts. They watched it. (291–293, S10)

Knowledge sharing has become a core element in enhancing learning within teams. The nursing students recognized the learning needs (e.g., lack of knowledge in a particular area) of other team members and played a collaborative role in facilitating the learning of other students. They demonstrated their competence as team players and fueled the team toward success.

There were some technical terms that we were not familiar with. Therefore, the students from the discipline who knew these terms would try to explain them. For example, when a social work student found that a technical term was quite foreign, we would try to explain it in a simplified manner so that he could grasp the situation. (16–19, N8)

I [a nursing student] could use my own knowledge, such as explaining the terms to a social work student or medical student. They understood much more after my explanation. (85–87, N9)

There was also a strong sense of professionalism and self-identity among the students. They noted that they could be professional representatives and enabled the team to learn more about their own profession. Hence, they were sufficiently recognized and valued to be given a case to manage during their participation.

There were a few reasons for my wanting to join those activities. First, I wanted to know more about other professions. Second, I wanted to make new friends. Third, if I had not attended, other students might not have been able to learn about my profession. Therefore, I had to go to allow others to know us. Hence, I wanted to have more participants from my discipline. (259–262, S11)

I also think that my self-esteem was boosted because [the students in my group] did not know the answers to some questions. This proves that physiotherapists have a role in managing cases. We play a valuable role in the healthcare teams. (81–82, S15)

### Professional-Related Outcome of Interprofessional Learning

The interactions among the students enriched their understanding of other professions in the healthcare system. This allowed them to gain insight into the strengths and limitations of their own and other healthcare professions.

#### Knowing Others

The nursing students indicated that their traditional training seldom mentioned the detailed roles of other healthcare professionals. This IPTBL course provided them with a better understanding of the differences and roles of different healthcare professionals.

The roles of physicians and social workers are clearer to me now. In our nursing school lectures, we mainly focused on topics related to nursing; the nursing students are told only those patients with psychological problems needed to be referred to other professionals, such as mental health professionals or medical social workers. After participating in this course…we have a better understanding of the job and roles of medical social workers for pediatric patients with developmental delays. (93–103, N19)

We know more about a social worker's work after attending this course. In the past, we believed that social workers only dealt with financial or family problems. Therefore, we referred these matters to social workers. We now have a better understanding of social workers. Instead of handling only the referral work, they also play a role in arranging the escort of patients to a hospital. We have now discovered some of the specific scopes of their work. (5–10, N4)

The students were also able to determine the limitations of other professions in caring for patients. They gained more confidence in working with others to apply the relevant knowledge and skills to help patients in real life.

I have learned that medical students play a very important role in making a diagnosis, but they may not have a strong foundation in rehabilitation concepts, such as the various outcome measures that are commonly used in clinics. It strengthened my belief that although doctors are certainly very important, they are not the only medical professionals who help patients. Other disciplines also play unique roles in treating patients. (36–42, S18)

One physiotherapy student pointed out that the IPTBL activity gave him an opportunity to learn the rationale behind the clinical judgments of other healthcare professions:

I learned that other professionals would consider different aspects of our case vignette. For example, I was quite impressed by a multiple-choice question that asked whether an MRI or CT scan should be performed for the condition... and I realized that they [the radiology students] did not simply memorize the scanning procedure. They understood the indications of the different scans and the implications of the various findings on the images. This activity improved my understanding of the other professions. (158–163, S11)

#### Knowing of Self

Most of the students increased their awareness of their roles and professional attributes during the discussion of different ways of learning in their professions.

The medical and social work students were asked about the characteristics of [nursing students'] clinical placements and teaching formats. They did not understand us. They said, “Really? Nursing students have to start working and perform hands-on techniques during clinical placements? We just focus on studying cases and verbally reporting what could be done.” The nursing students need more practical work during our clinical placement than the other students. We shared our clinical experiences with them. (3645, N4)

I told [the other students] that the nursing students needed to communicate with our patients and ask about their medical history. This was because we needed to fulfill the school's requirements for studying clinical cases. We asked our patients about the psychosocial aspects of their condition, and about their relationships with their families. We are also concerned about their relationships with neighbors if a patient is living alone. We told other students about what we performed during our clinical placements. (273–285, N20)

This makes me understand that every profession has its focus. Maybe physiotherapy emphasizes MSK [musculoskeletal] health, walking, balance, and so on. (6–8, S13)

### Patient-Related Outcome of Interprofessional Learning

The students treasured this learning activity because they could learn about different perspectives on caring for patients. They understood more about providing holistic care for their patients through effective interactions with different healthcare professionals.

#### An Expanded Perspective on Holistic Care

The students recognized that their patient management plans or approaches were only related to their own professional interests. One of the barriers in healthcare communication is the distinct professional position in the healthcare system. Both nursing and physiotherapy students learned that working with other healthcare students as a team might improve their communication skills. A collaborative interdisciplinary approach can be an effective way to yield optimal patient outcomes.

Situations for a specific disease need to be dealt with using an interdisciplinary approach to ensure that the outcome is best for the patient. For some chronic diseases, if the home environment of the patient is unsatisfactory, we may need other specialists such as social workers to assist us. In terms of communication skills, a nurse may not be as good as a social worker. As such, social workers may be more suited to understand the needs of patients. When we interview our patients, we may pay more attention to their biological needs. However, social workers may focus more on aspects related to patients' daily lives. In this learning activity, when different disciplines communicated with each other, I found that we could help by considering patients' conditions from different perspectives. Also, communicating with other disciplines in the workplace means that we can use our complementary expertise. We can better integrate our knowledge to help our patients. (71–83, N8)

We know how to support and provide more comprehensive services to the same patient by working on one case. In reality, we rarely encounter patients managed by a single medical profession. It is possible that different professionals cooperate with each other to benefit patients. I gained considerable experience from participating in this activity. (85–88, S10)

#### Exploring Care Options

The nursing students indicated that their learning experiences from a diverse group of healthcare students expanded their knowledge about different care options for their patients in the future. Team learning can broaden the knowledge of the students of other disciplines, which can help in their future patient care in various clinical settings.

The course is certainly useful for my work. For me, being a nurse or case manager, my work should always be patient centered. It is necessary for me to know what happens to my patients from admission to discharge so that I can make appropriate referrals to other disciplines. In this way, we can keep the different disciplines informed regarding the patient's condition and the discharge plan. (111–117 N9)

## Discussion

Our findings suggest that the undergraduate nursing and physiotherapy students gained a positive learning experience through this IPTBL activity. The students were able to build up their working relationships through active interactions and knowledge sharing. These findings are consistent with those of previous studies (5, 20) in which the recognition and development of a trustful relationship were found to allow people to understand one another (21) and form fundamental interprofessional relationships among themselves (22).

The descriptions of the students of their learning experiences suggest that they engaged in the process of self-awareness of their own profession. This is a critical point in the caring profession because the discovery of oneself is a continuous process for improvement (23). The Johari Window model, developed by Luft and Ingham (24), comprises four distinct panes of a window for reflecting on one's self-awareness in relation to others. The panes are “open” (known to the self and others), “blind” (not known to the self but known to others), “hidden” (known to self but not others), and “unknown” (not known to the self or others). Consistent with this model, the participating students were ready to open and disclose information and experiences to other team members during the activity in the open pane. Some students sought information and received constructive feedback from their team members, which was novel. This experience might have enlarged their open area into a blind pane, which might have led to a self-discovery of their own limitations in scaling down the unknown area. Furthermore, the self-reflection of their own roles and responsibilities during this activity eventually helped the students uncover the hidden pane. The design of the interprofessional learning activity enabled the students to work as a team and motivated them to learn from one another.

Professional socialization is a vital aspect of the development of undergraduate healthcare students. It is described as an internalization process to develop a professional identity (25). When students learn from and with other healthcare students through assimilation and accommodation processes, they improve not only their knowledge transfer but also enhance their understanding of the working culture and the nature of their own profession by comparing themselves with other healthcare students. This self-recognition of their own values as a profession can enable the students to complement one another while working as a team.

An understanding of different professional roles in a healthcare system can optimize collaboration in the workplace (Marian et al., 2021). The students in our study recognized that the existing professional training could not satisfy their needs because their understanding of other healthcare professional roles was insufficient. They believed that the adaptation of interprofessional education could eliminate this boundary. The students revealed that their management could involve different dimensions of care, thus, providing a more holistic and comprehensive plan for patients (26) after their improved understanding of other healthcare professionals. Holistic care can be easily attained using this collaborative approach.

Although interprofessional education has been widely adopted in most European countries, this teaching approach is relatively new to the health and social care curriculum in Hong Kong (17). However, the students in our study had positive learning experiences and expressed great support for this activity. The findings substantiate the need to equip the teaching staff to integrate IPTBL into a new pedagogy for healthcare students in Hong Kong.

Our research, as with any other studies, has various constraints. First, the assessment of the students may be reinforced *via* triangulation and follow-up to determine the long-term impact of the program on learners. Additional accurate statistics on the impact of IPTBL may be gathered once learners begin pursuing their careers. How they regard interdisciplinary cooperation in delivering care to service users seems to be a more accurate indication of its effectiveness, which might be interpreted as an impact of IPTBL. It is recommended that additional research address such issues and develop methods for delivering IPTBL that consider the constraints of our study. Despite these shortcomings, this study offers a framework for creating, executing, and assessing an IPE program, utilizing TBL as the teaching methodology for a large group of individuals enrolled in the undergraduate health and community care courses.

## Conclusions

This study showed that interprofessional education provides an important vehicle for undergraduate students to explore and develop their professional values. Using the team-based learning approach, the students were able to understand the importance of communication and collaboration in their future work. Future studies should investigate the experiences of IPTBL activities from the perspective of the teachers. Interprofessional team-based learning can enhance the learning experience of undergraduate students in healthcare education. Students develop confidence in their knowledge and communication skills through interprofessional team-based learning in trusting and complementary relationships. In addition, this study demonstrated a harmonious learning experience among undergraduate nursing and physiotherapy students. By using this IPE learning platform, the students had the opportunity to explore their own roles and understand their professional limitations and equip themselves to be more competent in collaborative work in caring for their patients in the future, hence, improving the patient outcomes. The study informs educational practice by showing the value of integrating IPE into undergraduate curricula of different health professions in the future.

## Data Availability Statement

The original contributions presented in the study are included in the article/supplementary files, further inquiries can be directed to the corresponding author/s.

## Ethics Statement

The studies involving human participants were reviewed and approved by The Hong Kong Polytechnic University. The patients/participants provided their written informed consent to participate in this study.

## Author Contributions

JH assisted to carry out the interviews. FW supervised the project and the findings of this work. JH, AW, and FW analysis of the results. VS, AC, and PT provided comment and clear suggestions. All authors discussed the results and contributed to the final manuscript.

## Funding

This work was supported by the University Grant Council, Special funding for teaching and learning (No. 89HX) and Faculty Dean's Reserve Research Grant (No. 1-ZVKV).

## Conflict of Interest

The authors declare that the research was conducted in the absence of any commercial or financial relationships that could be construed as a potential conflict of interest.

## Publisher's Note

All claims expressed in this article are solely those of the authors and do not necessarily represent those of their affiliated organizations, or those of the publisher, the editors and the reviewers. Any product that may be evaluated in this article, or claim that may be made by its manufacturer, is not guaranteed or endorsed by the publisher.
